# Pig Exposure and Health Outcomes in Hospitalized Infectious Disease Patients in Vietnam

**DOI:** 10.1007/s10393-019-01460-0

**Published:** 2019-12-16

**Authors:** Gail Robertson, Meghan Perry, Phat Voong Vinh, Dung Tran Thi Ngoc, Tam Pham Thi Thanh, Phuc Tran My, Huong Dang Thao, Maia Rabaa, Stephen Baker, Mark Woolhouse, the VIZIONS consortium

**Affiliations:** 1 School of Mathematics, James Clerk Maxwell Building, King’s Buildings, University of Edinburgh, Edinburgh, UK; 2 Epidemiology Research Group, King’s Buildings, University of Edinburgh, Edinburgh, UK; 3 The Hospital for Tropical Diseases, Wellcome Trust Major Overseas Programme, Oxford University Clinical Research Unit, Ho Chi Minh City, Vietnam; 4 Centre for Tropical Medicine and Global Health, Nuffield Department of Clinical Medicine, Oxford University, Oxford, UK; 5 Usher Institute of Population Health Sciences and Informatics, Ashworth Laboratories, King’s Buildings, University of Edinburgh, Edinburgh, UK

**Keywords:** Zoonosis, *Escherichia coli*, *Shigella*, Disease of unknown origin, Pigs, Vietnam, Southeast Asia

## Abstract

Many infectious diseases have a zoonotic origin, and several have had major public health implications. Contact with animals is a known risk factor for zoonotic infections, although there are limited data on disease symptoms and pathogens associated with contact with different animal species. The rise in pig production in Southeast Asia has contributed to the emergence and re-emergence of zoonotic infections caused by contact with pigs and pig products. To compare the symptom and pathogen profiles of hospitalized patients with and without pig contact, we collected data on disease symptoms, infecting pathogens, and animal contact behaviour from patients attending six hospitals across Vietnam between 2012 and 2016. Patients who had previous contact with pigs were more likely to have enteric disease than respiratory or central nervous system infections and were more likely to grow *Escherichia coli* and *Shigella* from stool culture than those without pig contact. Patients with enteric infections who kept pigs were also more likely to have a disease of unknown origin. Public health initiatives that account for differences in animal contact behaviours and offer more comprehensive diagnostics in high-risk individuals are needed if emergence and re-emergence of zoonotic disease is to be monitored and prevented.

**Electronic supplementary material:**

The online version of this article (10.1007/s10393-019-01460-0) contains supplementary material, which is available to authorized users.

## Introduction

Zoonotic disease is an ongoing global public health concern. Various environmental and demographic factors have been implicated in the spread of zoonotic diseases, including environmental degradation, increases in human and livestock populations (Karesh et al. 2012), and humans having sustained close contact with animals (Angulo et al. 2006, Paige et al. 2014). The incidence of zoonotic disease is likely to be higher in regions where there is greater opportunity for contact between humans and livestock or wildlife, which may occur through human activity or lack of adequate biosecurity (Karesh et al. 2012). There is a need to increase the detection of zoonotic transmission events and identify risk factors associated with such events, especially in regions where human/animal contact is more common (Molyneux et al. 2011, Morse et al. 2012).

Southeast Asia is recognized as a high-risk region for zoonotic disease due to high human and livestock population densities as well as widespread behavioural and cultural practices facilitating close and sustained contact between humans and animals (Jones et al. 2008). Due to their size, ease of keeping, and the growing popularity of pork, pigs are one of the most widely kept livestock species in Southeast Asia (Huynh et al. 2007). In Vietnam, pigs are an important meat source with > 98% of households reporting eating pork (Dinh et al. 2005, Wertheim et al. 2009). Some traditional dishes in Vietnam use raw or undercooked pig meat and products (Takahashi et al. 2000, Rabaa et al. 2015), which has been identified as a potential source of foodborne zoonoses (Conlan et al. 2011). Although various studies have identified pig contact as a risk factor for certain zoonotic pathogens, such as *Streptococcus suis* (Wertheim et al. 2009), little is known about the nature and the extent of pig contact in Southeast Asian countries and the disease syndromes and pathogens associated with such contact.

Diagnosing zoonotic disease quickly is vital to identify the source of infection and reduce the risk of future spillover events. Contact with pigs has been linked to the emergence of new zoonotic pathogens in Southeast Asia (e.g. Nipah virus) as well as with outbreaks of common endemic pathogens (e.g. Japanese encephalitis virus, *S. suis,* and *Salmonella*); hence, identifying symptoms and pathogens associated with this behaviour may assist clinicians in identifying patients likely to be involved in outbreaks of zoonotic disease and help guide public health policies. Here, we aimed to identify disease syndromes and pathogens associated with contact with pigs in Vietnam. We utilized data collected as a component of the Vietnam Initiative on Zoonotic Infections (VIZIONS) hospital-based surveillance project (Rabaa et al. 2015) to assess the extent of pig contact in Vietnam and to determine how such contact influences disease symptoms and severity in infectious disease patients admitted to hospital. Our specific aims were to: i) describe demographics and regional distribution of patients who had previous pig contact; ii) compare disease syndromes (enteric, respiratory, central nervous system infections (CNSIs)), pathogens, and hospital diagnoses of patients with and without previous pig contact; iii) determine whether patients with previous exposure to pigs had more severe disease than non-exposed patients. We expected patients with previous pig contact to be exposed to a greater number of unusual pathogens that are more difficult to diagnose than patients with no contact, and therefore be at greater risk of more severe disease due to more severe symptoms and lack of timely diagnosis. Other variables such as patient age, gender, and distance travelled from hospital may also affect disease severity, and these were controlled for using statistical methods.

## Methods

### The Vietnam Initiative on Zoonotic Infections (VIZIONS)

**Figure 1:**
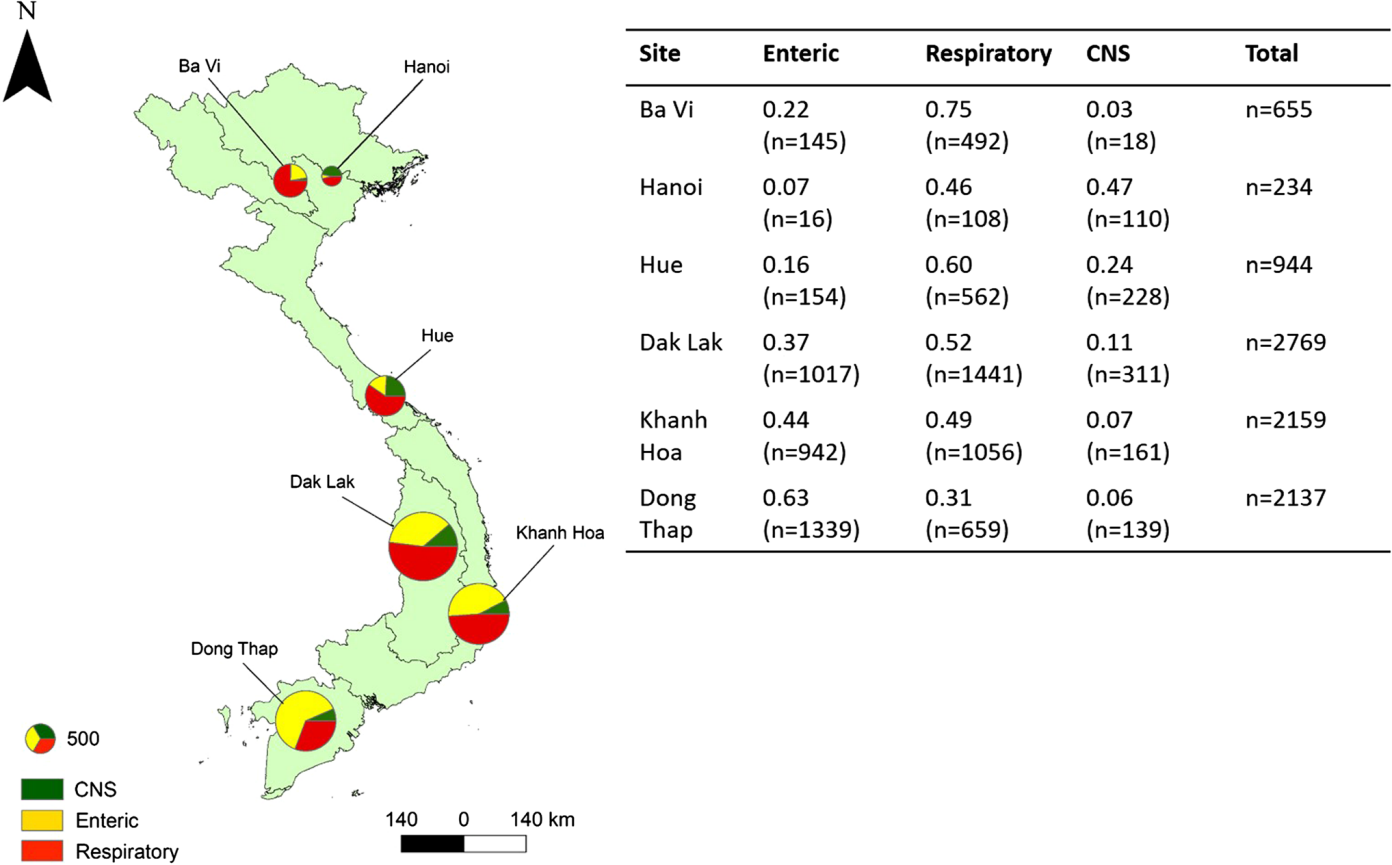
Proportions of patients with different syndromes admitted to six hospital sites across Vietnam. Size of pie charts correspond to relative number of patients with all three syndromes recruited at each hospital. Total *n* = 8898.

VIZIONS was a multidisciplinary Vietnam-based project established to increase our understanding of the origins and risks of zoonotic infections (Rabaa et al. 2015). Between March 2012 and August 2016, hospital admissions data were collected from six hospitals located in five regions of Vietnam (Fig. 1). Patients were recruited to the VIZIONS study shortly after admission to hospital with one of three defined clinical disease syndromes (enteric, respiratory, and central nervous system infections (CNSIs)) depending on primary symptoms on admission (Table 1). Written informed consent was obtained from all individual participants enrolled in the study. Patients whose symptoms were considered not to be associated with an infectious agent, who were not resident within the province of the hospital they were attending, or who had been previously hospitalized within 6 months were excluded.

**Table 1:** Inclusion and exclusion criteria used by attending physicians for recruiting patients to the VIZIONS study with each disease syndrome.

Enteric	Respiratory	CNS infections
*Inclusion criteria* Acute diarrhoeal infections (three or more loose stools or one bloody stool within a 24-h period) *Exclusion criteria* Patients with multiple complications unrelated to diarrhoeal disease or those with suspected antibiotic-related diarrhoea	*Inclusion criteria* Fever, or a history of fever over past 3 days. Must be under 55 years of age and have had respiratory symptoms for less than 7 days when admitted to hospital	*Inclusion criteria* At least 1 month old, fever or history of fever over the past 3 days. Presented with at least one of the following symptoms: headache, neck stiffness, altered consciousness, or focal neurological signs

On the day that patients were recruited, a member of VIZIONS staff distributed a questionnaire to each patient detailing demographics, symptoms, and behaviour prior to hospital admission (Table 2). For children and infants, the questionnaire was completed by a parent or guardian. Information regarding animal keeping or slaughtering was completed for the household in which the patient was living prior to becoming ill. Each questionnaire contained 22 to 24 questions for patients to complete (not including questions regarding which specific animal species patients had had contact with). Patients were asked to record the type of animals they had had contact with within two weeks of being admitted to hospital out of a list of 29 animals. To reduce the number of questions and increase accuracy of responses regarding animal or water source contact, we did not include questions on frequency of contact or timing of contact. Patients were able to complete questionnaires within 1 h. Questions on patient demographics, home address, symptoms, and behaviour were completed by patients, and these responses merged with hospital admission and discharge data, and results of patient sample testing (Table 2). Hospital discharge data were available for each patient after final outcome of illness was known. From March 2012 to August 2016, 3616 enteric (diarrhoea), 4326 respiratory, and 968 CNSI patients were recruited (*n* = 8910). Complete questionnaires were available for 8898 of those. Table S1 summarizes demographic and behavioural variables of interest for each hospital admission site.

**Table 2:** Information from hospital records and questionnaires completed by patients upon recruitment to VIZIONS.

Data	Enteric	Respiratory	CNSI
*Hospital admissions data*			
Hospital site (*n* = 6)	X	X	X
Date of admission	X	X	X
*Symptoms*			
Three or more days fever (yes/no)	X	X	X
Blood in stool (yes/no)	X		
Mucoid in stool (yes/no)	X		
Number of diarrhoea episodes	X		
Abdominal pain (yes/no)	X		
Muscle aches (yes/no)		X	
Any chronic respiratory illness (yes/no)		X	
HIV positive (yes/no)			X
*Questionnaire demographics*			
Age (years)	X	X	X
Gender (male/female)	X	X	X
Home location (address and spatial coordinates to commune level)	X	X	X
Distance of home to hospital (km)	X	X	X
*Questionnaire behaviour*			
Contact with patients with same syndrome (yes/no)	X	X	X
Water source (tap/bottled/pond/river/rainfall/well)	X	X	X
Animal contact (keep, slaughter, or eat/handle raw or undercooked meat, blood, or viscera) within two weeks of exhibiting signs of illness	X	X	X
List of animal species patient may have had contact with*	X	X	X
*Laboratory testing*			
Blood test results (including haemoglobin (g/dL); white blood cell count (10^9/L); platelet count (10^9/L); neutrophils count (%); lymphocytes count (%); eosinophils count (%))	X	X	X
Pathogen tested for**	X	X	X
*Hospital discharge data*			
Date of discharge	X	X	X
ICD10 discharge code and notes***	X	X	X
Length of stay in hospital (days)	X	X	X
Outcome^a^	X	X	X

Some questions regarding symptoms and medical history were only included in questionnaires for patients with specific disease syndromes. X denotes inclusion of a question in questionnaires distributed to patients with each syndrome

*Bamboo rat, bat, bear, buffalo, cat, cattle, chicken, civet, deer, dog, duck, goat, goose, jungle fowl, monkey, Muscovy duck, ornamental songbird, other wild bird, pangolin, pig, pigeon, porcupine, quail, rabbit, rat, sheep, squirrel, turkey, wild pig

**See Table S2

***As listed in International Statistical Classification of Diseases and Related Health Problems 10th Revision

^a^1 = Discharge with complete recovery, 2 = Discharge with incomplete recovery, 3 = Transferred to another hospital, 4 = Death/discharged to die, 5 = Discharged without permission, 6 = Unknown, 7 = Other

### Pathogen Detection and Hospital Diagnosis

Clinical specimens (faecal samples from enteric patients, sputum/nasal swabs from respiratory patients, cerebrospinal fluid from CNSI patients) were collected from patients on the day of recruitment to screen for pathogens predicted a priori to be the most common aetiological agent for each syndrome. Bacteria culture was performed in the laboratories of the collaborating hospitals; MacConkey agar was used to test for the presence/absence of *Escherichia coli* (hereafter *E. coli*) in stool samples; however, further tests to differentiate pathogenic and non-pathogenic strains of *E. coli* were not performed. Additional samples were shipped to Oxford University Clinical Research Unit (OUCRU) in Ho Chi Minh City where real-time polymerase chain reaction (qPCR) and enzyme-linked immunosorbent assays (ELISA) were used to screen for selected pathogens (Table S2, Table S3). Enteric samples also underwent routine microscopy to screen for parasitic infections. Patients in which no known pathogens were detected were defined as patients with a disease of unknown origin (DUO). Bacteria proved difficult to culture from nasal swabs; hence, the number of respiratory patients with DUOs was likely to be overestimated. At the conclusion of their time in hospital, the attending physician allocated each patient a clinical classification code (ICD10 code) describing primary disease classification according to the International Statistical Classification of Diseases 2010, which we used to represent their clinical diagnosis.

### Statistical Analyses

As patient home addresses (reported at the commune level) were located within a mean of 12.2 ± 0.3 km of their admission hospital (Table S1, Figure S1), we examined regional variation in pig contact by comparing behaviour of patients among hospital sites. Figure S1 shows that for some hospitals (Ba Vi, Hanoi, Dong Thap), patient addresses are clustered close to the hospitals, while at other sites, patient locations are spread over a wider area (Hue). This pattern may be explained by the number of alternative public hospitals available in each region (i.e. access to alternative hospitals may have been more restricted in the North Central region in which the Hue admission hospital is located, hence patients from a wider area attended). The pattern may also be explained by hospitals specializing in treating specific syndromes (e.g. CNSIs in Hanoi hospital) which attracts patients from further away.

We used several binomial generalized linear mixed models (GLMMs) to examine regional variation and demographics of patients with and without pig contact. Whether or not patients reported previous contact with pigs was included as the response variable, and demographic variables (such as gender and age) and hospital site and admission year were included as explanatory variables, depending on the specific question being addressed by the model. Disease syndrome (enteric, respiratory, or CNSI) was included as a random factor to account for variation in animal contact among patients with different syndromes and allow generalities of patients with all syndromes to be made. We used Chi-squared tests and Cramer’s V statistics to determine whether patients who had had contact with pigs were also more likely to have contact with other common livestock and pet species (cats, cattle, chickens, and dogs).

To examine possible associations between pig contact and disease syndrome, we used a multinomial logistic regression using the R package ‘nnet’ (Venables and Ripley 2002), in which disease syndrome was a three-level factor response variable. Whether or not a patient had previous pig contact was included as the explanatory variable of interest. The following variables were also included in the model: whether or not the patient had contact with other animal species (cats, cattle, chickens, and dogs), hospital admission site, distance between patient’s home address and admission hospital (km), age (adult or child (< 17 years old at time of admission)), gender, water source (natural (pond, river, rainfall, well) or unnatural (bottled or tap)), and year of admission. As there was a clear distinction between frequency of adult and child patients recruited, we included age as a two-level factor in models (78% of all patients were < 17 years old). We expected that affliction with a certain disease syndrome may be affected by various demographic and behavioural factors other than the variable of interest (pig contact). Previous studies have identified relationships between contact with specific animal species and disease syndrome (e.g. poultry contact and respiratory disease (Bridges et al. 2002)). To account for these relationships, we included contact with common animal species as a potential confounding variable in our model. We expected that distance between patients’ home address and admission hospital would also vary depending on disease syndrome patients were admitted with, for example patients with CNSIs may travel further to hospital as symptoms for this syndrome are more severe. Hence, distance between home address and hospital was also included in this model. Exploratory analysis showed that CNSIs were more frequently reported in patients > 20 years old and some age-related syndromes may be expected to be more frequent in specific genders; hence, age and gender were also included as confounding variables. As pathogens which cause enteric disease are often waterborne, we included water source in the model. As Hanoi hospital was located close to the hospital at Ba Vi, we merged data from these sites for the purpose of analysis. We used an ANOVA-based model selection procedure (Crawley 2013) to assess the importance of pig contact in explaining disease syndrome. Each variable was tested for significance using likelihood ratio tests (LRTs) (Fox and Weisberg 2011). The multinomial model was repeated to include different types of pig contacts (keeping, slaughtering, eating/handling raw pig meat, blood, or viscera).

We compared types of pathogens found in patients with and without pig contact using binomial GLMMs with hospital site as a random factor and whether or not a patient had contact with pigs as the response variable. Using a GLMM allowed us to account for variation in pig contact among patients in different hospital sites without estimating individual parameters for each site. As patients can test positive for more than one pathogen, we included each pathogen as a separate explanatory variable in the models, as well as potential confounders, patient age, gender, distance travelled to hospital, and admission year all of which we expected to affect pig contact behaviour of patients (i.e. older male patients who lived in rural areas may be more likely to have had contact with pigs). To compare hospital diagnoses in patients with and without pig contact, we used similar binomial GLMMs including ICD10 codes allocated to each patient as an explanatory variable. These analyses were carried out separately for different disease syndromes and types of pig contact behaviours.

Finally, we examined potential associations between pig contact and mortality risk and length of stay in hospital (days), the latter being a proxy for morbidity. We used GLMMs with whether or not a patient died in hospital and length of stay as response variables and whether or not a patient had contact with pigs as an explanatory variable, as well as potential confounding variables (gender, age, distance travelled to hospital, and admission year) which may also be expected to affect mortality and length of stay. Older patients who travelled further from hospital were expected to spend longer in hospital with greater risk of mortality. Mortality and morbidity in hospitals vary depending on year so admission year was also included as a confounding variable. Hospital site was included as a random factor in models. This analysis was repeated for each disease syndrome.

All analyses were implemented in R version 3.4.4 (R Core Development Team 2018).

## Results

### Demographics and Distribution of Patients With/Without Pig Contact

**Figure 2:**
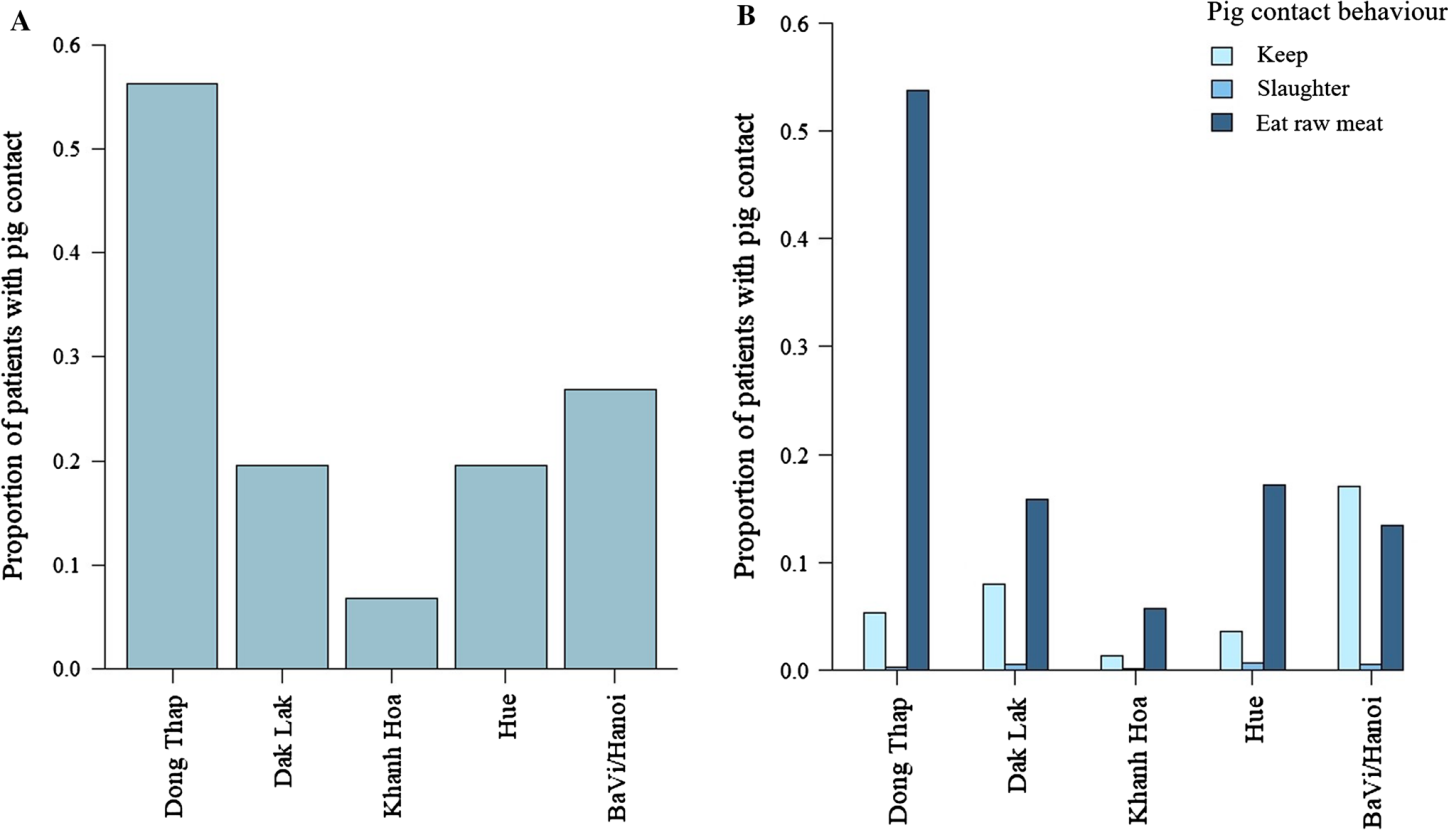
Proportions of patients admitted at each hospital site who A) had any contact with pigs (Dong Thap *n* = 1201, Dak Lak *n* = 543, Khanh Hoa *n* = 147, Hue *n* = 185, Ba Vi/Hanoi *n* = 239) and B) kept (Dong Thap *n* = 113, Dak Lak *n* = 221, Khanh Hoa *n* = 30, Hue *n* = 34, Ba Vi/Hanoi *n* = 151), slaughtered (Dong Thap *n* = 6, Dak Lak *n* = 15, Khanh Hoa *n* = 2, Hue *n* = 6, Ba Vi/Hanoi *n* = 5), or ate/handled raw meat, blood, or viscera from pigs (Dong Thap *n* = 1149, Dak Lak *n* = 439, Khanh Hoa *n* = 123, Hue *n* = 162, Ba Vi/Hanoi *n* = 120) (*n* = 8898).

The animal species with which the majority of patients had had contact were cats, cattle, chickens, dogs, and pigs (Figure S2); > 26% of all patients had had contact with pigs. Contact with pigs was most commonly associated with eating/handling raw pig meat, blood, or viscera; this was consistent across sites (with the exception of Ba Vi/Hanoi where keeping pigs was more common) (Fig. 2). The proportion of patients who had had contact with pigs varied significantly depending on admission hospital site (GLMM Wald test: *χ*_4_^2^ = 1083.2, *p *< 0.001, *n* = 8898; Table S1), with patients from Dong Thap the most likely to have had contact with pigs (log OR ± SE with Dong Thap as reference site: Dak Lak = − 1.58 ± 0.07, 95% confidence intervals (CIs) = − 1.71 to − 1.45; Hue = − 1.59 ± 0.10, 95% CIs = − 1.80 to − 1.39; Khanh Hoa = − 2.85 ± 0.10, 95% CIs = − 3.04 to − 2.66; Ba Vi/Hanoi = − 0.95 ± 0.09, 95% CIs = − 1.14 to − 0.77, Fig. 2). This result appears to be associated with the relatively high proportion of patients admitted in Dong Thap who had eaten/handled raw pig products (Fig. 2).

A greater proportion of female patients reported contact with pigs than male patients (GLMM Wald test: *χ*_1_^2^ = 30.6, *p *< 0.001, *n* = 8898; log OR ± SE: female patients = 0.28 ± 0.05, 95% CIs = 0.18–0.38, Figure S3), mainly from eating/handling raw pig products (25% of female and 20% of male patients had eaten/handled raw pig products). Older patients were significantly more likely to have had contact with pigs than younger patients (GLMM Wald test: *χ*_1_^2^ = 895.8, *p *< 0.001; log OR ± SE = 0.63 ± 0.02, 95% CIs = 0.59 to 0.67). The proportions of patients with pig contact also varied with the year of admission (Wald test: *χ*_4_^2^ = 105.2, *p *< 0.001, Figure S3). There was no significant difference in distance travelled to hospital for patients who did/did not have previous pig contact (Wald test: *p* = 0.49, *n* = 8898, Figure S3).

Patients who had had contact with pigs were also more likely to have had contact with cattle (Chi-squared test: *χ*_1_^2^ = 2268.8, *p *< 0.001, *n* = 8898), chickens (*χ*_1_^2^ = 2099.5, *p *< 0.001), dogs (*χ*_1_^2^ = 507.7, *p *< 0.001), and cats (*χ*_1_^2^ = 98.9, *p *< 0.001). Cramer’s V statistics suggested that the strongest associations were between contact with pigs and contact with cattle and chickens (Table S4, Figure S4).

### Disease Symptoms of Patients With/Without Pig Contact

**Figure 3:**
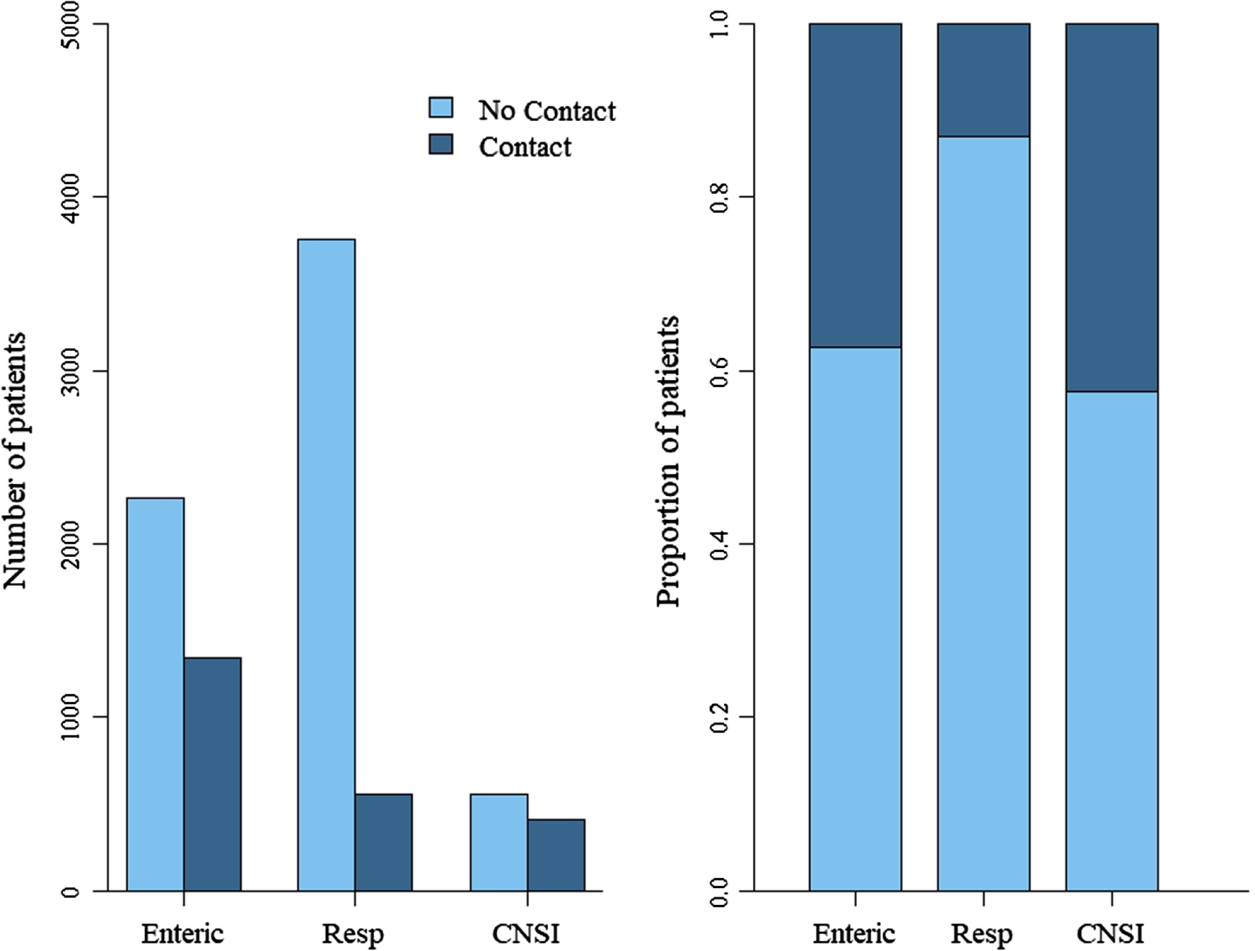
Numbers and proportions of patients who did/did not have contact with pigs prior to admission to hospital with different disease syndromes (enteric = 3613, respiratory = 4318, CNSI = 967).

Patients who had had contact with pigs presented with significantly different disease syndromes than patients without pig contact (Table 3, Fig. 3). Patients who had had contact with pigs were more likely to be admitted to hospital with an enteric disease than with other syndromes (Table 3). Specifically, patients who had eaten/handled raw pig products were more likely to have an enteric disease than other syndromes; this was less apparent for patients with other types of pig contact (Table S5).

**Table 3:** Coefficients and standard errors from a multinomial logistic regression examining effect of pig contact on disease syndrome accounting for variation in spatiotemporal and demographic factors.

	Respiratory	CNSI	df	LR *χ*^2^	*p* value
Site:	–	–	8	757.7	< 0.001*
Dong Thap	− 0.73 ± 0.05	− 1.18 ± 0.02	–	–	–
Hue	0.96 ± 0.04	1.79 ± 0.03	–	–	–
Khanh Hoa	− 0.45 ± 0.05	− 0.66 ± 0.03	–	–	–
Ba Vi/Hanoi	1.12 ± 0.05	0.86 ± 0.03	–	–	–
Distance from hospital (per 10 km)	− 0.05 ± 0.01	0.06 ± 0.01	2	75.5	< 0.001*
Gender	0.06 ± 0.05	0.60 ± 0.08	2	53.8	< 0.001*
Age	0.39 ± 0.06	2.18 ± 0.04	2	522.2	< 0.001*
Year of admission	− 0.28 ± 0.001	− 0.57 ± 0.001	2	195.3	< 0.001*
Water source	− 0.07 ± 0.05	− 0.65 ± 0.05	2	45.4	< 0.001*
Pig contact	− 1.36 ± 0.04	− 0.41 ± 0.04	2	291.0	< 0.001*
Cattle contact	0.16 ± 0.04	0.07 ± 0.03	2	2.8	0.25
Chicken contact	− 0.14 ± 0.06	− 0.14 ± 0.03	2	4.3	0.12
Dog contact	− 0.30 ± 0.05	− 0.26 ± 0.03	2	19.1	< 0.001*
Cat contact	0.20 ± 0.03	− 0.16 ± 0.02	2	9.0	0.011*

Results of likelihood ratio tests (LRTs) comparing models including and excluding each variable are displayed. Coefficients and standard errors are given in the multinomial logit scale. *represents a significant *p* value (< 0.05). Total *n* = 8898

Values displayed for site, gender, age, water source, and syndrome are given relative to Dak Lak, females, children, natural sources, and enteric syndrome, respectively. The first year of admission was 2012

### Pathogen Testing in Patients With/Without Pig Contact

Enteric patients who had previous contact with pigs were more likely to grow *E. coli* (any type) and *Shigella* in stool samples, but less likely to test positive for Astrovirus than patients who had not had recent pig contact (Table 4). Eating/handling raw pig products was associated with an increased risk of *Shigella* in enteric patients (Table S6), while keeping pigs was associated with an increased risk of *E. coli* and Adenovirus (Table S7). When models were repeated including cat, cattle, chicken, or dog contact as the response variable, eating/handling raw cattle or chicken meat was also associated with an increased likelihood of growing *Shigella* (cattle: *χ*_1_^2^ = 6.1, *p* = 0.01; chicken: *χ*_1_^2^ = 5.5, *p* = 0.02). No significant association with any enteric pathogen (including *E. coli* and Adenovirus) was found for patients who kept other animal species (cattle, chickens, dogs, or cats).

**Table 4:** Results of a binomial GLMM comparing types of pathogens of enteric patients who did/did not have pig contact (including hospital site as a random factor).

Variables	Coefficient	95% confidence levels (lower, upper)	LR *χ*^2^	df	*p* value
Adenovirus	0.21	(− 0.36, 0.77)	0.5	1	0.47
Aichivirus	0.39	(− 1.50, 2.28)	0.2	1	0.68
Astrovirus	− 0.74	(− 1.46, − 0.02)	4.1	1	0.04*
*E. coli*	0.32	(0.05, 0.59)	5.4	1	0.02*
Norovirus 2	0.07	(− 0.26, 0.39)	0.2	1	0.68
Rotavirus	− 0.17	(− 0.42, 0.09)	1.7	1	0.19
*Salmonella*	0.26	(− 0.48, 1.00)	0.5	1	0.49
*Shigella*	1.18	(0.35, 2.02)	7.7	1	0.006*
Sapovirus	0.12	(− 0.59, 0.84)	0.1	1	0.73
DUO	0.02	(− 0.34, 0.39)	0.02	1	0.90
Distance from hospital (per 10 km)	− 0.04	(− 0.11, 0.04)	0.9	1	0.33
Gender	− 0.06	(− 0.27, 0.14)	0.4	1	0.55
Age	− 2.71	(− 3.01, − 2.40)	297.0	1	< 0.001*
Year of admission	0.39	(0.30, 0.49)	62.0	1	< 0.001*

Results of Wald tests for each variable are displayed, as well as log odds ratio estimates and their 95% confidence intervals. Coefficients and 95% confidence intervals are from the global model. *represents a significant *p* value (< 0.05). Total *n* = 3615

Values displayed for gender and age are given relative to females and children, respectively. The first year of admission was 2012

Although we found that enteric patients who had previous contact with pigs were not more likely to have a DUO than patients who had not had pig contact (Table 4), patients who kept pigs were more likely to have a DUO than those who did not keep pigs (Table S7). This finding suggests that while pig contact in general had no effect on whether or not an enteric patient had a DUO, patients who had had close contact with live pigs were more likely to have a DUO than non-pig keepers. GLMMs which included keeping cats, cattle, chickens, or dogs as response variables rather than keeping pigs showed that this association was only apparent for enteric patients who kept pigs, and not other animal species (GLMM Wald test *p* values: cats = 0.26, cattle = 0.76, chickens = 0.49, dogs = 0.46).

No individual pathogens were positively associated with pig contact in respiratory or CNSI patients, but respiratory patients with pig contact were less likely to test positive for RSV (Table S8, Table S9), and no significant association between pig contact and the established meningitis pathogen *S. suis* was found for CNSI patients (Chi-squared test: *p* = 0.55, *n* = 836).

### Clinical Diagnosis of Patients With/Without Pig Contact

The ICD10 code that included *E. coli* infection (A04) was significantly more likely to be allocated to enteric patients who had had previous pig contact, and viral enteric infections (A08) were less likely to be allocated to patients with pig contact (Table 5). However, shigellosis (A03) was allocated less frequently in patients with pig contact, contrary to results of pathogen testing (Tables 4, 5). Enteric patients who tested positive for *E. coli* were significantly more likely to be allocated the A04 ICD10 code in comparison with patients who did not test positive for *E. coli* (Chi-squared test: *χ*_1_^2^ = 549.2, *p *< 0.001, *n* = 3615). No association was found between patients who tested positive for *Shigella* and the corresponding ICD10 code (*p* = 0.66). No enteric patients were allocated ICD10 codes for parasitic infections.

**Table 5:** Results of a binomial GLMM comparing ICD10 hospital codes of enteric patients who did/did not have pig contact (including hospital site as a random factor).

Variables	Coefficient	95% confidence levels (lower, upper)	LR *χ*^2^	df	*p* value
ICD10 code:	–	–	36.4	4	<0.001*
A03	− 0.85	(− 1.61, − 0.09)	–	–	–
A06	− 0.70	(− 2.14, 0.75)	–	–	–
A08	− 1.01	(− 1.37, − 0.66)	–	–	–
A09	− 0.27	(− 0.72, 0.18)	–	–	–
Distance from hospital (per 10 km)	− 0.06	(− 0.14, 0.03)	1.7	1	0.20
Gender	− 0.05	(− 0.27, 0.16)	0.2	1	0.63
Age	− 2.71	(− 3.03, − 2.40)	282.7	1	< 0.001*
Year of admission	0.41	(0.31, 0.52)	62.7	1	< 0.001*

Results of Wald tests for each variable are displayed, as well as log odds ratio estimates and their 95% confidence intervals. Coefficients and 95% confidence intervals are from the global model. Patients with the ICD10 code A05 (Other bacterial intoxications, not elsewhere classified) were excluded as < 10 patients were diagnosed with this code. *represents a significant *p* value (< 0.05). Total *n* = 3432

Values displayed for ICD10 code, gender, and age are given relative to A04 (‘other bacterial intestinal infections’ including *E.coli*), females, and children, respectively. A03 = ‘Shigellosis’; A06 = ‘Amoebiasis’; A08 = ‘Viral and other specified intestinal infections’; A09 = ‘Other gastroenteritis and colitis of infectious and unspecified origin’. The first year of admission was 2012

Hospital diagnoses differed significantly between respiratory patients who did/did not have previous pig contact (GLMM Wald test: *χ*_8_^2^ = 16.4, *p* = 0.04, *n* = 3860), with patients with pig contact more likely to be allocated ICD10 code for influenza (J11), although ICD10 codes were allocated based on symptoms and were not confirmed by molecular testing (Table S10). CNSI patients who had had pig contact were more likely to be diagnosed with bacterial meningitis (ICD10 code G01) and encephalitis (G04) (Table S11), despite a lack of association between pig contact and positive test results for *S. suis* (as shown above).

### Disease Severity in Patients With/Without Pig Contact

There was no association between length of time spent in hospital (days) and contact with pigs for enteric patients (GLMM Wald test: *p* = 0.66, *n* = 3613). Hospital mortality could not be examined for enteric patients as only five enteric patients died in hospital. There was no effect of pig contact on hospital mortality for respiratory or CNSI patients (respiratory: *p* = 0.46, *n* = 4205; CNSI: *p* = 0.39, *n* = 780). There was a significant effect of pig contact on length of stay for respiratory patients (Wald test: *χ*_1_^2^ = 6.18, *p* = 0.01, *n* = 4315): patients who had had contact with pigs spent comparatively less time in hospital than those who had not had pig contact (log OR ± SE = − 0.11 ± 0.04, 95% CIs = − 0.20 to -0.02). CNSI patients who had had contact with pigs spent similar amounts of time in hospital as those who had not had pig contact (Wald test: *p* = 0.31, *n* = 966).

## Discussion

This is the first large-scale study to assess the extent of prior pig contact in hospital patients in a Southeast Asian country and its association with infectious disease and health outcomes. Over 26% of patients reported pig contact in our study, the most common of which was eating/handling raw/undercooked pig products. Our analysis shows that type and frequency of pig contact varied significantly among hospital sites which were located in different regions around Vietnam. Contact by eating/handling raw pig products was most frequently reported by patients at the hospital in Dong Thap Province, while keeping pigs was most frequently reported by patients who were admitted to hospital in Hanoi/Ba Vi. These results correspond with regional pig density estimates in Vietnam, which are highest in the northeast around Hanoi as well as in the Mekong Delta region close to Dong Thap (Wertheim et al. 2009). Regional variation in type of contact may be explained by cultural differences and socioeconomic factors throughout Vietnam (Phuong et al. 2014).

Zoonotic and foodborne pathogens have been identified in pig production facilities in Vietnam, and the prevalence of such pathogens may be greater in smallholder pig farms (Tran et al. 2004, Carrique-Mas et al. 2014). Eating raw or undercooked pig products in traditional dishes, which occurs in Southeast Asian countries including Vietnam (Ho et al. 2011, Huong et al. 2014), increases potential for spread of zoonotic pathogens from pigs to humans (Wertheim et al. 2009, Carrique-Mas and Bryant 2013, Huong et al. 2014). Twenty-two percent of patients in our study reported this behaviour, similar to results of a previous study in Hanoi Province which reported that an average of 21% of rural and urban individuals ate raw pig products as a part of traditional dishes (Huong et al. 2014).

Patients with previous pig contact, especially eating/handling raw pig products, were more likely to be admitted to hospital with symptoms of enteric disease than respiratory disease or CNSIs, accounting for confounding variables such as age, gender, and year of admission to hospital. Diarrhoea is common in Vietnam, with community-based studies estimating a prevalence of approximately 1–3 cases per child per year (Carrique-Mas et al. 2014). However, the pathogens responsible for enteric illness in Vietnam often go undiagnosed due to the lack of access to healthcare and laboratory services (Kelly-Hope et al. 2007, Peeling and Mabey 2010). This study employed a range of pathogen detection methods (including PCR and culture) to compare pathogen and diagnostic profiles of patients who did and did not have contact with pigs. Patients with enteric disease who had previous contact with pigs were significantly more likely to test positive for *E. coli* and *Shigella* than patients without pig contact. No associations with pig contact were found for other enteric pathogens.

Shigellosis can cause severe diarrhoeal disease, especially in children and infants, and concerns regarding antimicrobial resistance have increased efforts to control *Shigella*, particularly in developing countries (Kotloff et al. 1999, Seidlein et al. 2006). Eating raw or undercooked meat is a recognized risk factor for *Shigella* infection, due to infected individuals contaminating foods in preparation for consumption (Bryan 1988), and eating/handling raw meat (from pigs as well as other animal species) was found to be positively associated with *Shigella* infection in our study. As 75% of patients admitted to hospital with enteric disease were under five years old, questionnaire responses referred to behaviours practiced by other members of the household. Lack of hygiene in food preparation and eating undercooked food are common risk factors for shigellosis which occurs in Vietnam (Kelly-Hope et al. 2007, Takanashi et al. 2009) and may explain the significant association between eating/handling raw meat (from pigs, cattle, or chickens) and *Shigella* infection. Increased awareness of the risk of acquiring *Shigella* from poor hygiene as well as consumption of raw or undercooked meat products may prevent future infections from this widespread pathogen.

Enteric patients who reported keeping pigs were more likely to test positive for *E. coli* than those who did not keep pigs. Although our study did not differentiate between pathogenic and non-pathogenic *E. coli* variants, patients who tested positive for *E. coli* were also more likely to be allocated the ICD10 code ‘A04’ which includes a diagnosis of pathogenic *E. coli* (‘bacterial intestinal infections’ including *E. coli*). Given that pigs are the one of the most commonly kept livestock species in Vietnam and Southeast Asia, keeping pigs or contact with environments used by pigs may be an important risk factor for infection with pathogenic *E. coli* (Kobayashi et al. 2003).

Our results also show that enteric patients who kept pigs were more likely to have a disease of unknown origin than those who did not keep pigs, but no such associations were found in enteric patients who kept other animal species. Diagnostic testing was more complete for patients with enteric syndrome (parasites were tested for as well as a variety of viruses and bacteria expected to cause enteric symptoms), and only 21% of all enteric patients had DUOs (compared with 33% and 68% of respiratory and CNSI patients, respectively). However, enteric patients who kept pigs were less often diagnosed than those who did not keep pigs (72% and 80%, respectively), possibly because of poorer testing for more specific zoonotic infections (e.g. Nipah virus, influenza, and *Trichinella*).

Patients with respiratory and CNSI syndromes showed no difference in types of pathogens infecting patients with and without pig contact. We expected an association between *S. suis* infection and pig contact, but no significant association was found. This may be due to difficulties in diagnosing pathogens causing CNSI using cerebrospinal fluid (Kotilainen et al. 1998, Rimerio et al. 2015); approximately 68% of CNSI patients were negative for all pathogens they were tested for. Swine influenza is another major concern to public health in Southeast Asia (Choi et al. 2011). Our study found no positive association between pig contact and respiratory syndrome; however, ICD10 codes specific for influenza were more likely to be allocated to patients with pig contact than patients who did not have contact with pigs.

Although patients with previous exposure to pigs tested positive for a different suite of pathogens than patients without pig contact, patients with pig contact did not spend longer in hospital or have an increased risk of mortality. Patients who kept pigs were more likely to have DUOs (which may have been more unusual pathogens not routinely tested for), but did not show signs of more serious disease; hence, contrary to expectations, undiagnosed pathogens in this patient group did not cause more severe disease than diagnosed pathogens.

As this is a large-scale questionnaire-based study, there are some limitations that must be highlighted. Firstly, pathogen testing for some syndromes (specifically CNSI) was limited due to difficulties in culturing and diagnosing pathogens from cerebrospinal fluid, making DUOs more frequently assigned to CNSI patients than patients with other syndromes. Secondly, questionnaire-based studies are subject to reporting bias and hospital patients are more likely to report unusual or risky behaviours prior to becoming ill (Nieuwenhuijsen 2005). Lastly, our study was carried out on hospital patients and exposure to pigs should be examined in the general population of Vietnam to determine the extent of this behaviour.

## Conclusion

This large-scale epidemiological study is the first to document the extent of pig contact in hospital patients in Vietnam and identify symptom and pathogen profiles of patients who reported this behaviour. Contact with pigs is common in Vietnam and Southeast Asia due to increasing demand for pork and the consequent increase in both small- and large-scale pig farming. Our study found that patients who had contact with pigs were more likely to exhibit symptoms of enteric disease, and that pathogen and diagnostic profiles differed in enteric patients with and without pig contact. Enteric patients with pig contact were more likely to test positive for the zoonotic pathogen *E. coli*, as well as the foodborne pathogen *Shigella* than those without pig contact, and patients who kept pigs were more likely to have a DUO than those who did not. Given the prevalence of zoonotic pathogens in both small- and large-scale animal production systems in Southeast Asia, as well as in raw/undercooked meat (Van et al. 2012), our results highlight the need for public health initiatives which control zoonotic and foodborne pathogens throughout the food supply chain and educate individuals regarding safe food preparation practices. Our study also highlights the need for more comprehensive diagnostics in hospital patients with close animal contact who may be at an increased risk of pathogens not usually tested for during routine diagnostic procedures. We recommend the development of programmes to increase awareness of risk factors for zoonotic disease in Vietnam, offering guidelines on how high-risk individuals can minimize their risk of infection by employing safe food preparation and increased hygiene practices. Further research to determine the prevalence of severe zoonotic infections (e.g. Nipah virus and pathogenic *E. coli*) in Vietnam and the extent to which pig contact is a risk factor for these infections is also necessary.

## Electronic supplementary material

Below is the link to the electronic supplementary material.

Supplementary material 1 (DOCX 3776 kb)
