# Supplementary material for: Pig Exposure and Health Outcomes in Hospitalized Infectious Disease Patients in Vietnam
Source: Ecohealth. 2019 Dec 16;17(1):28–40. doi: 10.1007/s10393-019-01460-0 (PMC7109191; doi:10.1007/s10393-019-01460-0)
Supplement: Supplementary file 1 — Supplementary material 1 (DOCX 3776 kb) [file 10393_2019_1460_MOESM1_ESM.docx]

**Supplementary material**

**Tables**

**Table S1.** Summary statistics (mean ± SE, median, proportion, N) for demographics, disease characteristics, and pig contact behavior of patients at each hospital admission site.

|  | **Ba Vi** | | | **Hanoi** | | | **Hue** | | | | **Dak Lak** | | | **Khanh Hoa** | | | **Dong Thap** | | | **All sites** | | |
| --- | --- | --- | --- | --- | --- | --- | --- | --- | --- | --- | --- | --- | --- | --- | --- | --- | --- | --- | --- | --- | --- | --- |
|  | Mean ± SE, median | Prop | N | Mean ± SE, median | Prop | N | | Mean ± SE, median | Prop | N | Mean ± SE, median | Prop | N | Mean ± SE, median | Prop | N | Mean ± SE, median | Prop | N | Mean ± SE, median | Prop | N |
| Age | 2.9 ± 0.3, 1 | - | 655 | 29.3 ± 1.2, 28 | - | 234 | | 11.9 ± 0.7, 2 | - | 944 | 7.5 ± 0.3, 1 | - | 2769 | 12.7 ± 0.4, 2 | - | 2159 | 15.9 ± 0.6, 2 | - | 2137 | 11.5 ± 0.2, 2 | - | 8898 |
| Gender (male) | - | 0.58 | 380 | - | 0.60 | 141 | | - | 0.62 | 585 | - | 0.59 | 1633 | - | 0.53 | 1145 | - | 0.53 | 1130 | - | 0.56 | 8898 |
| Distance to hospital (km) | 3.9 ± 0.1, 3.7 | - | 655 | 34.2 ± 2.7, 17.1 | - | 234 | | 22.0 ± 1.3, 7.3 | - | 944 | 15.7 ± 0.5, 8.1 | - | 2769 | 8.0 ± 0.6, 3.2 | - | 2159 | 7.8 ± 0.1, 5.5 | - | 2137 | 12.2 ± 0.3, 5.5 | - | 8898 |
| Length of stay (days) | 8.2 ± 0.5, 7 | - | 654 | 10.0 ± 0.8, 6 | - | 234 | | 8.3 ± 0.4, 5 | - | 944 | 5.9 ± 0.2, 4 | - | 2766 | 4.2 ± 0.1, 3 | - | 2159 | 4.9 ± 0.1, 4 | - | 2136 | 5.8 ± 0.1, 4 | - | 8893 |
| Disease syndrome | - | Enteric = 0.22;  Resp = 0.75;  CNSI = 0.03 | 145; 492; 18 | - | Enteric = 0.07;  Resp = 0.46;  CNSI = 0.47 | 16; 108; 110 | | - | Enteric = 0.16;  Resp = 0.60;  CNSI = 0.24 | 154; 562; 228 | - | Enteric = 0.37;  Resp = 0.52;  CNSI = 0.11 | 1017; 1441; 311 | - | Enteric = 0.44;  Resp = 0.49;  CNSI = 0.07 | 942; 1056; 161 | - | Enteric = 0.63;  Resp = 0.31;  CNSI = 0.06 | 1339; 659; 139 | - | Enteric = 0.41;  Resp = 0.49;  CNSI = 0.10 | 8898 |
| Pig contact (Yes) | - | 0.19 | 127 | - | 0.48 | 112 | | - | 0.20 | 185 | - | 0.20 | 543 | - | 0.07 | 147 | - | 0.56 | 1201 | - | 0.26 | 8898 |
| Keep pigs (Yes) | - | 0.18 | 121 | - | 0.13 | 30 | | - | 0.04 | 34 | - | 0.10 | 221 | - | 0.01 | 30 | - | 0.05 | 113 | - | 0.06 | 8898 |
| Slaughter pigs (Yes) | - | 0.005 | 3 | - | 0.009 | 2 | | - | 0.006 | 6 | - | 0.005 | 15 | - | 0.0009 | 2 | - | 0.003 | 6 | - | 0.004 | 8898 |
| Eat/handle raw pork (Yes) | - | 0.02 | 16 | - | 0.44 | 104 | | - | 0.17 | 162 | - | 0.16 | 439 | - | 0.06 | 123 | - | 0.54 | 1149 | - | 0.22 | 8898 |

**Table S2.** Bacteria and viruses tested for using bacteriological and viral diagnostic procedures (including qPCR and ELISA).

| **Enteric** | | **Respiratory** | | **CNSI** | |
| --- | --- | --- | --- | --- | --- |
| **Bacteria** | **Viruses** | **Bacteria** | **Viruses** | **Bacteria** | **Viruses** |
| *Acinetobacter nosocomialis* | Rotavirus A | *Leuconostoc spp* | Influenza A | *Acinetobacter baumannii* | Enterovirus |
| *Acinetobacter tandoii* | Norovirus 1 | *Abiotrophia adiacens* | Influenza B | *Alloiococcus otilis* | Varicella zoster virus |
| *Aeromonas salmonicida* | Norovirus 2 | *Acinetobacter baumannii* | Respiratory syncytial virus | *Burkholderia cepacia* | Dengue virus |
| *AeromoNegs hydrophyla* | Aichivirus | *Aeromonas hydrophia* | Enterovirus | *Cryptococcus neoformans* | Japanese encephalitis virus |
| *Bacillus subtilis* | Adenovirus | *Candida albicans* | Adenovirus | *Escherichia coli* (any type) | Herpes simplex virus |
| *Campylobacter spp* | Sapovirus | *Chryseomonas luteola* | Rhinovirus | *Enterococcus faecalis* |  |
| *Candida albicans* | Astrovirus | *Haemophilus influenzae* | Metapneumovirus | *Haemophylus influenzae* |  |
| *Citrobacter freundii* |  | *Klebseilla oxytoca* | Parainfluenza virus (1 to 4) | *Klebsiella pneumoniae* |  |
| *Citrobacter youngae* |  | *Klebsiella pneumoniae* | Coronavirus 1-2 | *Moraxella spp* |  |
| *Comamonas kerstersii* |  | *Moraxella spp* | Parechovirus | *Salmonella spp* |  |
| *Delftia acidovorans* |  | *Neisseria flavescens* | Bocavirus | *Staphylococcus cohnii* |  |
| *Escherichia coli* (any type) |  | *Proteus spp* |  | *Staphylococcus epidermidis* |  |
| *Edwardsiella tarda* |  | *Pseudomonas aeruginosa* |  | *Staphylococcus haemolyticus* |  |
| *Entercoccus avium* |  | *Pseudomonas putida* |  | *Staphylococcus spp* |  |
| *Enterobacter asburiae* |  | *Staphylococcus aureus* |  | *Streptococcus mitis* |  |
| *Enterobacter cloacae* |  | *Stenotrophomonas maltophilia* |  | *Streptococcus pneumoniae* |  |
| *Enterococcus faecalis* |  | *Streptococcus mitis 1* |  | *Streptococcus pneumoniae* |  |
| *Enterococcus faecium* |  | *Streptococcus parasanguinis* |  | *Streptococcus pyogenes* |  |
| *Klebseilla pneumoniae* |  | *Streptococcus pneumoniae* |  | *Streptococcus suis* |  |
| *Kluyvera georgiana* |  | *Streptococcus salivarius* |  | *Streptococcus suis II* |  |
| *Morganella morganii* |  | *Streptococcus sangius* |  |  |  |
| *Proteus mirabilis* |  | *Streptococcus α* |  |  |  |
| *Proteus penneri* |  |  |  |  |  |
| *Proteus vulgaris* |  |  |  |  |  |
| *Pseudomonas aeruginosa* |  |  |  |  |  |
| *Pseudomonas putida* |  |  |  |  |  |
| *Salmonella spp* |  |  |  |  |  |
| *Serratia liquefecient* |  |  |  |  |  |
| *Shigella flexneri* |  |  |  |  |  |
| *Shigella sonnei* |  |  |  |  |  |
| *Staphylococcus aureus* |  |  |  |  |  |
| *Staphylococcus sciuri* |  |  |  |  |  |
| *Staphylococcus warneri* |  |  |  |  |  |
| *Vibrio albensis* |  |  |  |  |  |
| *Vibrio cholerae* |  |  |  |  |  |

**Table S3.** Bacteria and viruses responsible for enteric, respiratory, and CNSI syndromes tested for using bacteriological and viral diagnostic procedures (including qPCR and ELISA). Percentages of patients who tested positive for each pathogen are displayed. Percentages of respiratory patients with each bacterial pathogen were calculated from 227 patients with sputum samples. Pathogens most commonly identified in VIZIONS hospital patients and included in further analyses are highlighted in bold. Note that some patients tested positive for more than one pathogen.

| **Syndrome** | **Bacteria** | **Viruses** |
| --- | --- | --- |
| Enteric (n = 3,615) | ***Escherichia coli* (42.6%)**  ***Salmonella*  (1.5%)**  ***Shigella*  (1.5%)**  *Citriobacter spp* (0.4%)  *Campylobacter*  (0.3%)  *Enterobacter spp* (0.3%)  *Acinetobacter*  (0.1%) | **Rotavirus A (37.3%)**  **Norovirus 2 (15.2%)**  **Adenovirus (2.9%)**  **Astrovirus (2.8%)**  **Sapovirus (2.0%)**  **Aichivirus (0.4%)**  Norovirus 1 (0.1%) |
| **Unknown = 21%** |  |  |
| Respiratory (n = 227) | *Streptococcus spp* (14.5%)  *Klebsiella spp* (7.9%)  *Candida spp* (1.3%) | **Respiratory syncytial virus (22.7%)**  **Rhinovirus (12.7%)**  **Influenza A (11.5%)** |
|  |  | **Parainfluenza virus (1 to 4) (7.6%)**  **Bocavirus (7.3%)**  **Adenovirus (6.1%)**  **Metapneumovirus (4.1%)**  **Coronavirus 1-2 (3.7%)**  **Enterovirus (3.6%)**  **Influenza B (2.5%)** |
|  |  | Parechovirus (0.6%) |
| **Unknown = 33%** |  |  |
| CNSI (n = 836) | ***Streptococcus suis* (10.2%)**  ***Streptococcus pneumoniae* (6.5%)**  *Staphylococcus spp* (1.0%)  *Neisseria meningitis* (0.8%)  *Haemophilus influenzae* type B (0.3%)  *Cryptococcus spp* (0.2%) | **Japanese encephalitis virus (7.6%)**  **Enterovirus (3.3%)**  **Dengue virus (2.1%)**  **Herpes simplex virus (1.4%)**  Varicella zoster virus (0.6%) |
| **Unknown = 68%** |  |  |

**Table S4.** Cramer’s V statistics showing strength of association between different types of animal contact.

|  | **Pig contact** | **Cattle contact** | **Chicken contact** | **Dog contact** | **Cat contact** |
| --- | --- | --- | --- | --- | --- |
| **Pig contact** | 1.00 |  |  |  |  |
| **Cattle contact** | 0.51 | 1.00 |  |  |  |
| **Chicken contact** | 0.49 | 0.33 | 1.00 |  |  |
| **Dog contact** | 0.24 | 0.13 | 0.31 | 1.00 |  |
| **Cat contact** | 0.11 | 0.09 | 0.18 | 0.57 | 1.00 |

**Table S5.** Coefficients and standard errors from a multinomial logistic regression examining effect of different types of pig contact (keep, slaughter, or eat/handle raw pig meat, blood, or viscera) on disease syndrome accounting for variation in spatiotemporal and demographic factors. Results of likelihood ratio tests (LRTs) comparing models including and excluding each variable are displayed. Coefficients are given in the multinomial logit scale. * represents a significant p-value (<0.05). Total n = 8,898.

|  | **Respiratory** | **CNSI** | **df** | **LR Χ^2^** | **p-value** |
| --- | --- | --- | --- | --- | --- |
| Site: | - | - | 8 | 658.5 | <0.001 * |
| Dong Thap | -0.62 ± 0.05 | -1.07 ± 0.04 | - | - | - |
| Hue | 0.99 ± 0.04 | 1.85 ± 0.03 | - | - | - |
| Khanh Hoa | -0.49 ± 0.05 | -0.65 ± 0.05 | - | - | - |
| BaVi/Hanoi | 1.00 ± 0.05 | 0.81 ± 0.03 | - | - | - |
| Distance from hospital (per 10 km) | -0.05 ± 0.01 | 0.06 ± 0.01 | 2 | 73.0 | <0.001 * |
| Gender | 0.05 ± 0.05 | 0.59 ± 0.08 | 2 | 51.9 | <0.001 * |
| Age | 0.53 ± 0.05 | 2.24 ± 0.04 | 2 | 497.6 | <0.001 * |
| Year of admission | -0.24 ± 0.001 | -0.54 ± 0.001 | 2 | 158.3 | <0.001 * |
| Watersource | -0.03 ± 0.05 | -0.62 ± 0.05 | 2 | 42.1 | <0.001 * |
| Keep pig | -0.09 ± 0.02 | 0.41 ± 0.02 | 2 | 11.5 | 0.003 * |
| Slaughter pig | 1.16 ± 0.001 | 1.14 ± 0.001 | 2 | 5.3 | 0.07 |
| Eat/handle pig meat | -1.74 ± 0.05 | -0.61 ± 0.05 | 2 | 409.9 | <0.001 * |
| Cattle contact | 0.24 ± 0.05 | 0.09 ± 0.03 | 2 | 5.6 | 0.06 |
| Chicken contact | -0.16 ± 0.06 | -0.19 ± 0.03 | 2 | 5.6 | 0.06 |
| Dog contact | -0.33 ± 0.05 | -0.30 ± 0.05 | 2 | 22.6 | <0.001 * |
| Cat contact | 0.18 ± 0.03 | -0.20 ± 0.03 | 2 | 8.8 | 0.012 * |

Values displayed for site, gender, age, water source, and syndrome are given relative to Dak Lak, females, children, natural sources, and enteric syndrome respectively. The first year of admission was 2012.

| **Variables** | **Coefficient** | **95% confidence levels (lower, upper)** | **LR Χ^2^** | **df** | **p-value** |
| --- | --- | --- | --- | --- | --- |
| Adenovirus | 0.01 | (-0.59, 0.62) | 0.002 | 1 | 0.97 |
| Aichivirus | 0.77 | (-1.19, 2.73) | 0.6 | 1 | 0.44 |
| Astrovirus | -0.72 | (-1.52, 0.07) | 3.2 | 1 | 0.07 |
| *E. coli* | 0.23 | (-0.06, 0.51) | 2.4 | 1 | 0.12 |
| Norovirus 2 | 0.09 | (-0.27, 0.45) | 0.2 | 1 | 0.62 |
| Rotavirus | -0.19 | (-0.45, 0.08) | 1.8 | 1 | 0.17 |
| *Salmonella* | -0.16 | (-0.96, 0.65) | 0.1 | 1 | 0.70 |
| *Shigella* | 0.96 | (0.10, 1.82) | 4.7 | 1 | 0.03 * |
| DUO | -0.02 | (-0.42, 0.38) | 0.01 | 1 | 0.92 |
| Distance from hospital (per 10 km) | -0.07 | (-0.16, 0.03) | 1.8 | 1 | 0.18 |
| Gender | -0.07 | (-0.29, 0.15) | 0.3 | 1 | 0.56 |
| Age | -3.17 | (-3.52, -2.83) | 319.6 | 1 | <0.001 * |
| Year of admission | 0.53 | (0.43, 0.64) | 96.1 | 1 | <0.001 * |

**Table S6.** Results of a binomial GLMM comparing types of pathogens of enteric patients who did/did not eat/handle raw pig meat (including hospital site as a random factor). Results of Wald tests for each variable are displayed, as well as log odds ratio estimates and their 95% confidence intervals. As only 15 patients who ate/handled raw pig meat tested positive for Sapovirus this pathogen was excluded from the model. Coefficients and 95% confidence intervals are from the global model. * represents a significant p-value (<0.05). Total n = 3,615.

Values displayed for gender and age are given relative to females and children respectively. The first year of admission was 2012.

**Table S7.** Results of a binomial GLMM comparing types of pathogens of enteric patients who did/did not keep pigs (including hospital site as a random factor). Results of Wald tests for each variable are displayed, as well as log odds ratio estimates and their 95% confidence intervals. Coefficients and 95% confidence intervals are from the global model. As no patients who kept pigs tested positive for Aichivirus this pathogen was excluded from the model. * represents a significant p-value (<0.05). Total n = 3,615.

| **Variables** | **Coefficient** | **95% confidence levels (lower, upper)** | **LR Χ^2^** | **df** | **p-value** |
| --- | --- | --- | --- | --- | --- |
| Adenovirus | 0.89 | (0.13, 1.65) | 5.3 | 1 | 0.02 * |
| Astrovirus | -0.60 | (-2.03, 0.84) | 0.7 | 1 | 0.42 |
| *E. coli* | 0.92 | (0.42, 1.41) | 13.0 | 1 | <0.001 * |
| Norovirus 2 | 0.03 | (-0.51, 0.57) | 0.01 | 1 | 0.91 |
| Rotavirus | 0.15 | (-0.26, 0.57) | 0.5 | 1 | 0.47 |
| Salmonella | 0.94 | (-0.04, 1.92) | 3.5 | 1 | 0.06 |
| *Shigella* | 1.09 | (-0.04, 2.23) | 3.6 | 1 | 0.06 |
| *Sapovirus* | 0.46 | (-0.63, 1.55) | 0.68 | 1 | 0.41 |
| DUO | 0.69 | (0.12, 1.25) | 5.6 | 1 | 0.02 * |
| Distance from hospital (per 10 km) | 0.03 | (-0.02, 0.08) | 1.1 | 1 | 0.30 |
| Gender | 0.01 | (-0.30, 0.33) | 0.01 | 1 | 0.93 |
| Age | -1.17 | (-1.54, -0.79) | 37.7 | 1 | <0.001 * |
| Year of admission | -0.43 | (-0.59, -0.27) | 28.3 | 1 | <0.001 * |

Values displayed for gender and age are given relative to females and children respectively. The first year of admission was 2012.

**Table S8.** Results of a binomial GLMM comparing types of pathogens of respiratory patients who did/did not have pig contact (including hospital site as a random factor). Results of Wald tests for each variable are displayed, as well as log odds ratio estimates and their 95% confidence intervals. Coefficients and 95% confidence intervals are from the global model. * represents a significant p-value (<0.05). Total n = 4,326.

| **Variables** | **Coefficient** | **95% confidence levels (lower, upper)** | **LR Χ^2^** | **df** | **p-value** |
| --- | --- | --- | --- | --- | --- |
| Adenovirus | -0.30 | (-1.19, 0.58) | 0.5 | 1 | 0.50 |
| Bocavirus | -0.15 | (-0.96, 0.67) | 0.1 | 1 | 0.72 |
| Coronavirus | -0.92 | (-2.29, 0.43) | 1.8 | 1 | 0.18 |
| Enterovirus | -0.59 | (-1.96, 0.77) | 0.7 | 1 | 0.39 |
| Influenza A | -0.61 | (-1.44, 0.22) | 2.1 | 1 | 0.15 |
| Influenza B | -0.01 | (-1.01, 0.98) | 0.001 | 1 | 0.98 |
| Metapneumovirus | 0.25 | (-0.79, 1.29) | 0.2 | 1 | 0.64 |
| Parainfluenza virus | -0.66 | (-1.53, 0.22) | 2.2 | 1 | 0.14 |
| Rhinovirus | -0.67 | (-1.52, 0.18) | 2.4 | 1 | 0.12 |
| RSV | -0.86 | (-1.64, -0.08) | 4.7 | 1 | 0.03 * |
| DUO | -0.41 | (-1.19, 0.36) | 1.1 | 1 | 0.30 |
| Distance from hospital (per 10 km) | -0.15 | (-0.61, 0.30) | 0.4 | 1 | 0.51 |
| Gender | -0.21 | (-0.42, 0.004) | 3.7 | 1 | 0.05 * |
| Age | -2.75 | (-3.05, -2.46) | 342.4 | 1 | <0.001 * |
| Year of admission | -1.04 | (-1.19, -0.90) | 202.7 | 1 | <0.001 * |

Values displayed for gender and age are given relative to females and children respectively. The first year of admission was 2012.

**Table S9.** Results of a binomial GLMM comparing types of pathogens of CNSI patients who did/did not have pig contact (including hospital site as a random factor). Results of Wald tests for each variable are displayed, as well as log odds ratio estimates and their 95% confidence intervals. Coefficients and 95% confidence intervals are from the global model. * represents a significant p-value (<0.05). Total n = 836.

| **Variables** | **Coefficient** | **95% confidence levels (lower, upper)** | **LR Χ^2^** | **df** | **p-value** |
| --- | --- | --- | --- | --- | --- |
| Dengue virus | 0.36 | (-0.73, 1.44) | 0.4 | 1 | 0.52 |
| Enterovirus | -0.76 | (-2.40, 0.88) | 0.8 | 1 | 0.37 |
| Herpes simplex | 1.07 | (-0.76, 2.90) | 1.3 | 1 | 0.25 |
| Japenese encephalitis | 0.45 | (-0.28, 1.17) | 1.4 | 1 | 0.23 |
| *S.pneumoniae* | 0.80 | (0.02, 1.58) | 4.0 | 1 | 0.05 * |
| *S.suis* | -0.22 | (-0.85, 0.42) | 0.4 | 1 | 0.50 |
| DUO | 0.11 | (-0.47, 0.69) | 0.2 | 1 | 0.71 |
| Distance from hospital (per 10 km) | 0.08 | (0.02, 0.13) | 6.4 | 1 | 0.01 * |
| Gender | 0.07 | (-0.25, 0.40) | 0.2 | 1 | 0.66 |
| Age | -1.53 | (-1.88, -1.19) | 77.5 | 1 | <0.001 * |
| Year of admission | -0.03 | (-0.22, 0.16) | 0.1 | 1 | 0.78 |

Values displayed for gender and age are given relative to females and children respectively. The first year of admission was 2012.

**Table S10.** Results of a binomial GLMM comparing ICD10 hospital codes of respiratory patients who did/did not have pig contact (including hospital site as a random factor). Results of Wald tests for each variable are displayed, as well as log odds ratio estimates and their 95% confidence intervals. Coefficients and 95% confidence intervals are from the global model. * represents a significant p-value (<0.05). Total n = 3,860.

| **Variables** | **Coefficient** | **95% confidence levels (lower, upper)** | **LR Χ^2^** | **df** | **p-value** |
| --- | --- | --- | --- | --- | --- |
| ICD10 code: |  |  | 16.4 | 1 | 0.04 * |
| J02 | -0.02 | (-0.62, 0.58) | - | - | - |
| J03 | 0.20 | (-0.75, 1.15) | - | - | - |
| J04 | -0.68 | (-1.75, 0.39) | - | - | - |
| J06 | 0.24 | (-0.32, 0.81) | - | - | - |
| J11 | 1.13 | (0.34, 1.93) | - | - | - |
| J16 | -0.06 | (-0.59, 0.46) | - | - | - |
| J20 | -0.31 | (-0.76, 0.13) | - | - | - |
| J21 | 0.20 | (-0.30, 0.71) | - | - | - |
| Distance from hospital (10km) | -0.005 | (-0.05, 0.04) | 0.1 | 1 | 0.80 |
| Gender | -0.16 | (-0.40, 0.07) | 1.9 | 1 | 0.17 |
| Age | -3.07 | (-3.51, -2.62) | 183.4 | 1 | <0.001 * |
| Year of admission | -1.12 | (-1.28, -0.96) | 190.5 | 1 | <0.001 * |

Values displayed for ICD10 code, gender and age are given relative to J18 (‘Pneumonia’), females and children respectively. J02 = ‘Acute pharyngitis’; J03 = ‘Acute tonsillitis’; J04 = ‘Acute laryngitis and tracheitis’; J06 = ‘Acute upper respiratory infections of multiple and unspecified sites’; J11 = ‘Influenza’; J16 = ‘Pneumonia due to other infectious organisms’; J20 = ‘Acute bronchitis’; J21 = ‘Acute bronchiolitis’. The first year of admission was 2012.

| **Variables** | **Coefficient** | **95% confidence levels (lower, upper)** | **LR Χ^2^** | **df** | **p-value** |
| --- | --- | --- | --- | --- | --- |
| ICD10 code: | - | - | 13.9 | 3 | 0.003 * |
| G01 | 1.14 | (0.35, 1.92) | - | - | - |
| G03 | -0.21 | (-1.56, 1.15) | - | - | - |
| G04 | 1.56 | (0.32, 2.80) | - | - | - |
| Distance from hospital (per 10 km) | 0.11 | (-0.01, 0.23) | 3.4 | 1 | 0.06 |
| Gender | 0.15 | (-0.34, 0.63) | 0.3 | 1 | 0.56 |
| Age | -1.94 | (-2.44, -1.44) | 58.0 | 1 | <0.001 * |
| Year of admission | -0.07 | (-0.36, 0.23) | 0.2 | 1 | 0.66 |

**Table S11.** Results of a binomial GLMM comparing ICD10 hospital codes of CNSI patients who did/did not have pig contact (including hospital site as a random factor). Results of Wald tests for each variable are displayed, as well as log odds ratio estimates and their 95% confidence intervals. Coefficients and 95% confidence intervals are from the global model. * represents a significant p-value (<0.05). Total n = 424.

Values displayed for ICD10 code, gender, and age are given relative to G00 (‘Bacterial meningitis, not elsewhere classified’), females, and children respectively. G01 = ‘Meningitis in bacterial diseases classified elsewhere’; G03 = ‘Meningitis due to other and unspecified causes’; G04 = ‘Encephalitis, myelitis and encephalomyelitis’. The first year of admission was 2012.

**Figures**

**
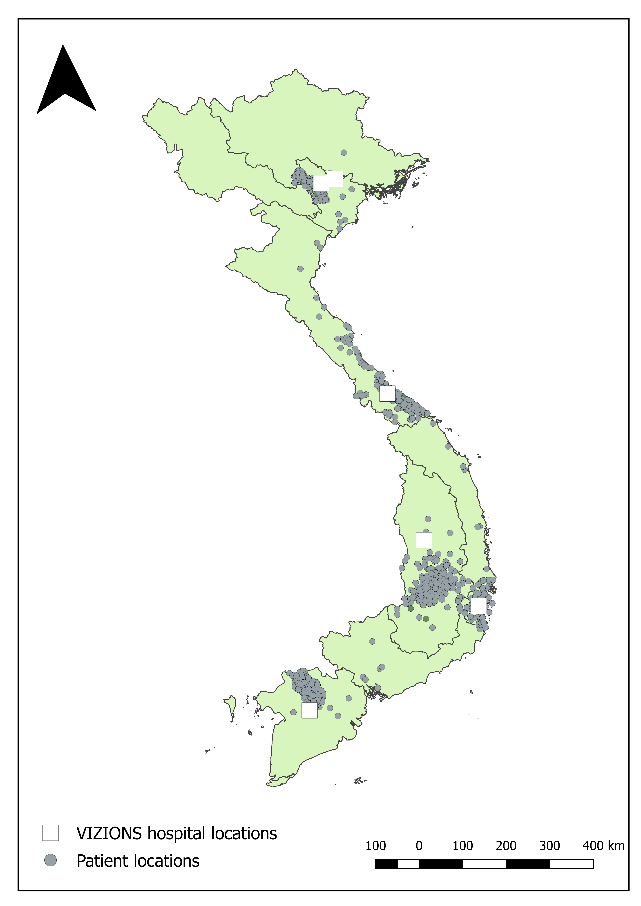
**

**Figure S1.** Location of patient home addresses (at commune level) in relation to admission hospital (n = 8,898).


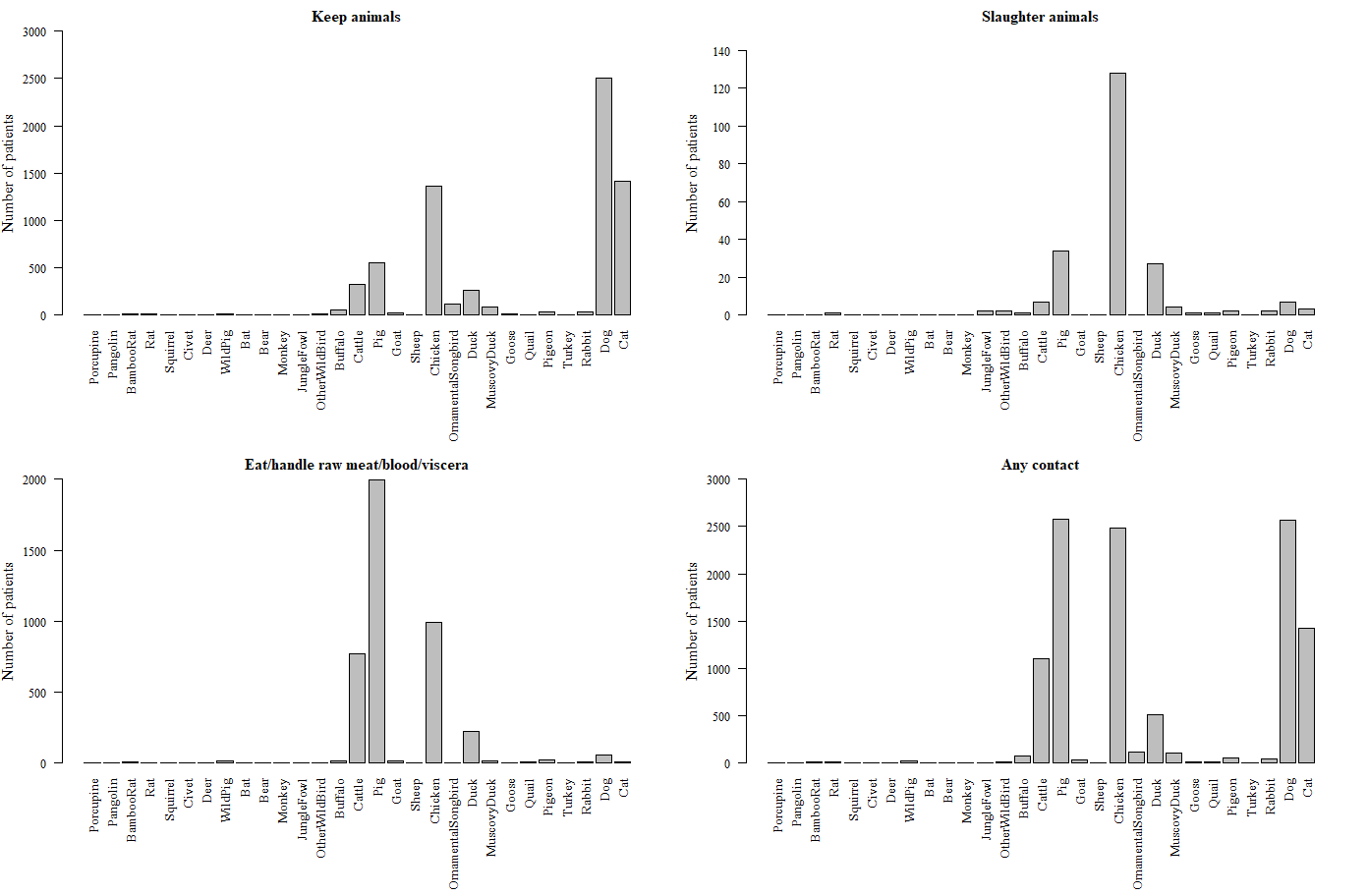


**Figure S2.** Numbers of patients who had contact with different animal species before admission to hospital (n = 8,898).


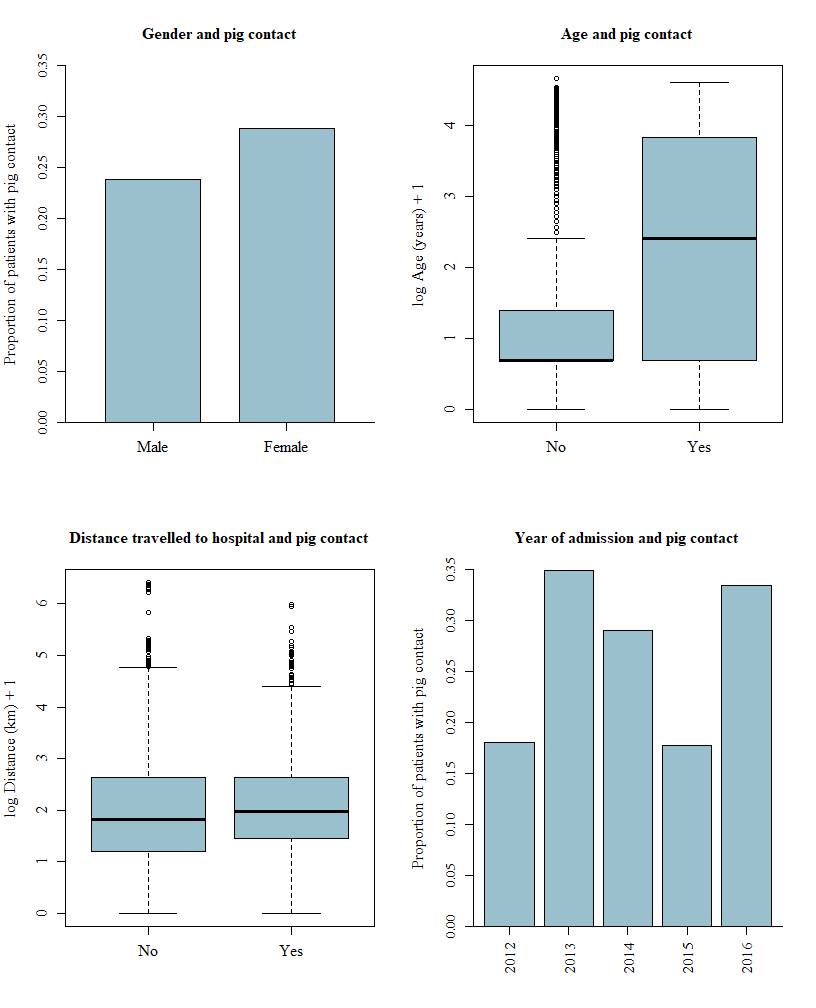


**Figure S3.** Plots showing associations between patient demographics and pig contact (n = 8,898).


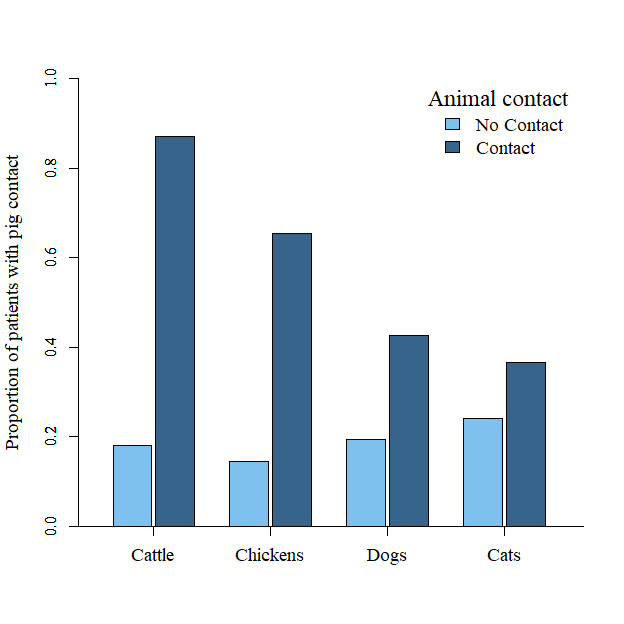


**Figure S4.** Proportions of patients with previous contact with pigs who did/did not have contact with other animal species (n = 8,898).
